# Oncologic Thermoradiotherapy: Need for Evidence, Harmonisation, and Innovation

**DOI:** 10.3390/cancers14102418

**Published:** 2022-05-13

**Authors:** Stephan Bodis, Pirus Ghadjar, Gerard van Rhoon

**Affiliations:** 1 Foundation for Research on Information Technologies in Society (IT’IS), 8004 Zürich, Switzerland; 2 Department of Radiation Oncology, University Hospital Zurich, 8032 Zürich, Switzerland; 3 Department Radiation Oncology, Charité–Universitätsmedizin Berlin, Corporate Member of Freie Universität Berlin and Humboldt-Universität zu Berlin, Augustenburger Platz 1, 13353 Berlin, Germany; pirus.ghadjar@charite.de; 4 Department of Radiotherapy, Erasmus MC Cancer Institute, University Medical Center, 3015 GD Rotterdam, The Netherlands; g.c.vanrhoon@erasmusmc.nl

## Evidence, Harmonisation and Innovation

The road of acceptance of oncologic thermotherapy/hyperthermia as a synergistic modality in combination with standard oncologic therapies is still bumpy. This is partially due to lack of evidence from international, multicentric, randomized clinical trials combined with biologic/pharmacologic systemic therapy and/or radiotherapy and/or surgery including a long term follow up.

Despite valid data and numerous publications over many decades, the lack of level I evidence for clinical trials, the lack of a thermotherapy/hyperthermia glossaries understood by all stakeholders with defined technical terms, and a widening technology gap between available and desirable hard-software components created a lukewarm climate for acceptance and reimbursement of thermotherapy/hyperthermia by most national health authorities and national health care insurances. Due to recently published randomized studies, a series of published meta-analysis and the first draft of a thermotherapy/hyperthermia glossary this field is moving forward.

Novel international research networks jointly by academia and industry (e.g., HYPERBOOST (https://www.hyperboost.eu/, last accessed on 2 May 2022); an EU horizon 2020 multimillion educational grant for PhDs) in the areas of AI/IT, biology, physics and technology are strengthening this joint effort substantially. We do hope that this special edition of Cancers can support these running, joint efforts.

## Evidence

In this special issue new evidence is presented. In a long term retrospective study of preoperative chemoradiation plus deep hyperthermia Schemm et al. [1]. reports an encouraging level in five-year survival with better RFS for patients reaching T50 temperatures above 39.9 °C. The impact of a high thermal dose was also found in the retrospective study on RT+HT for prostate cancer by Nakahare et al. [2]: CEM43T90 > 7 min. predicted improved biochemical disease-free survival. The paper by Minnaar et al. [3] performed a two- to three-year follow-up on the results of the South African Phase III Trial comparing chemoradiotherapy with and without modulated Electro-Hyperthermia (mEHT) and found a beneficial effect on the two- to three-year survival survival for patients with locally advanced cervical cancer treated by chemoradiation plus mEHT. The retrospective study by Lee et al. [4] evaluated the results of neoadjuvant 40 Gy plus mEHT followed by surgery in patients with advanced rectal cancer and observed an indication for longer survival in patients treated with mEHT energy above 3800 kJ.

The review by Szasz [5] discusses whether heterogenous heating is beneficial over homogenous heating. On the other hand, the review by Dewhirst et al. [6] emphasizes the importance of hyperthermia to improve perfusion and long duration reoxygenation (i.e., 24–48 h post hyperthermia).

For sure integration of mechanistical and pre-clinical immunology research data will further strengthen insights into biological mechanisms, increase understanding of the different patterns of in vivo tumor responses, and promote clinical trials integrating immunology in the field of oncologic thermotherapy. In this issue Sengedorj et al. [7] demonstrate that HT combined with RT changes the immunophenotype of breast cancer cells and upregulates immune suppressive immune checkpoint molecules. Therefore, adding an immune checkpoint inhibitor to combined HT-RT should be further explored as another potential clinical benefit.

## Harmonization

This issue contributes to further define the role of thermoradiotherapy. For instance, the review by Datta et al. [8] discusses the potential role of hyperthermia as a potential game-changer in the management of cancer in low-middle-income group countries. It reflects on different hyperthermia technologies that are available including annular-phased array systems for regional hyperthermia as well as capacitive hyperthermia systems without and with the use of amplitude modulation [1,2,3,4]. As each technology involves advantages and disadvantages, it is most important to consciously use it for appropriate clinical indications, preferably within prospective clinical trials. It is not primarily the choice of technology but rather the commonly observed unreasonable and mostly undocumented use of the method as explained by Ademaj et al. [9], that has dragged the collective success of thermoradiotherapy. An excellent example on how to introduce hyperthermia in a harmonized way at a national level is presented by Stutz et al. [10]. By introducing the Swiss Hyperthermia Network (https://www.ksa.ch/zentren-kliniken/radio-onkologie/leistungsangebot/swiss-hyperthermia-network, last accessed on 2 May 2022), hyperthermia treatment has been made available at a national level for all Swiss inhabitants enabling at the same time a platform to discuss with the Swiss Federal Office of Public Health (https://www.bag.admin.ch/bag/en/home.html, last accessed on 2 May 2022) proper implementation of reimbursement for hyperthermia treatment of selected evidence-based indications. The data provided in this issue support the use of different hyperthermia technologies for different well described clinical scenarios and will therefore improve the overall clinical acceptance of thermoradiotherapy in oncology.

## Innovation

This special issue is a scholarly example of innovation (i.e., defined as the successive introduction of new ideas, devices, and methods to generate improved output and value). The various contributions provide numerous suggestions to innovate thermotherapy, aiming at improved efficiency of combined thermoradiotherapy. The papers by Schemm et al. [1] and Nakahara et al. [2] are new support of the existence of a thermal dose effect relationship and is another confirmation for the need to always strive for the highest quality assurance and control for optimal treatment outcome. As reported by various papers improved efficiency can be achieved by innovating the heating and improved patient selection to fit with the correct level (complexity) of technology (Kroesen et al. [11], Poni et al. [12], Androulakis et al. [13]) enhanced understanding of the biological principles, either by experimental or clinical research (Dewhirst et al. [6], Sengedorj et al. [7]) or through building new more advanced biological models (Scheidegger et al. [14]) and supported by adequate computer modelling to predict the temperature distribution in the tissue (Kok et al. [15]). At the same time the contribution of Ademaj et al. [9] makes it crystal clear that to better understand which mechanisms are dominant to maximize treatment outcome during the clinical application of thermoradiotherapy, we still have a world to gain in accurate and complete documentation of the quality of the thermal therapy delivered to the patient. Improved documentation will open the gate way for further exploitation of thermal therapy using new technology to guide treatment quality and hence making thermal therapy more effective and efficient. Both items are crucial to increase the wider acceptance of thermal therapy by the oncological community and ultimately to bring the benefits of thermal therapy to the patients in all countries.

In this issue we find contributions from Africa, America, Asia, and Europe. Beside local, regional and national efforts we also need to strengthen international platforms and networks of oncologic thermorediotherapy. This is a must for a better global exchange of knowledge and information transfer. These oncologic thermo-therapy networks will further stimulate innovations, both for HI and LMI countries, strengthen harmonization including a well understood and accepted “vocabulary for thermotherapy”, improve acceptance of health care authorities and the public opinion, regulatory processes and QA. And, last but not least, all of these efforts must always be based on evidence.

## References

[1] Schem B.-C., Pfeffer F., Ott M.A., Wiig J.N., Sletteskog N., Frøystein T., Myklebust M.P., Leh S., Dahl O., Mella O. (2022). Long-Term Outcome in a Phase II Study of Regional Hyperthermia Added to Preoperative Radiochemotherapy in Locally Advanced and Recurrent Rectal Adenocarcinomas. Cancers.

[2] Nakahara S., Ohguri T., Kakinouchi S., Itamura H., Morisaki T., Tani S., Yahara K., Fujimoto N. (2022). Intensity-Modulated Radiotherapy with Regional Hyperthermia for High-Risk Localized Prostate Carcinoma. Cancers.

[3] Minnaar C.A., Maposa I., Kotzen J.A., Baeyens A. (2022). Effects of Modulated Electro-Hyperthermia (mEHT) on Two and Three Year Survival of Locally Advanced Cervical Cancer Patients. Cancers.

[4] Lee Y., Kim S., Cha H., Han J.H., Choi H.J., Go E., You S.H. (2022). Long-Term Feasibility of 13.56 MHz Modulated Electro-Hyperthermia-Based Preoperative Thermoradiochemotherapy in Locally Advanced Rectal Cancer. Cancers.

[5] Szasz A. (2022). Heterogeneous Heat Absorption Is Complementary to Radiotherapy. Cancers.

[6] Dewhirst M.W., Oleson J.R., Kirkpatrick J., Secomb T.W. (2022). Accurate Three-Dimensional Thermal Dosimetry and Assessment of Physiologic Response Are Essential for Optimizing Thermoradiotherapy. Cancers.

[7] Sengedorj A., Hader M., Heger L., Frey B., Dudziak D., Fietkau R., Ott O.J., Scheidegger S., Barba S.M., Gaipl U.S. (2022). The Effect of Hyperthermia and Radiotherapy Sequence on Cancer Cell Death and the Immune Phenotype of Breast Cancer Cells. Cancers.

[8] Datta N.R., Jain B.M., Mathi Z., Datta S., Johari S., Singh A.R., Kalbande P., Kale P., Shivkumar V., Bodis S. (2022). Hyperthermia: A Potential Game-Changer in the Management of Cancers in Low-Middle-Income Group Countries. Cancers.

[9] Ademaj A., Veltsista D.P., Ghadjar P., Marder D., Oberacker E., Ott O.J., Wust P., Puric E., Hälg R.A., Rogers S. (2022). Clinical Evidence for Thermometric Parameters to Guide Hyperthermia Treatment. Cancers.

[10] Stutz E., Puric E., Ademaj A., Künzi A., Krcek R., Timm O., Marder D., Notter M., Rogers S., Bodis S. (2022). Present Practice of Radiative Deep Hyperthermia in Combination with Radiotherapy in Switzerland. Cancers.

[11] Kroesen M., van Holthe N., Sumser K., Chitu D., Vernhout R., Verduijn G., Franckena M., Hardillo J., van Rhoon G., Paulides M. (2021). Feasibility, SAR Distribution, and Clinical Outcome upon Reirradiation and Deep Hyperthermia Using the Hypercollar3D in Head and Neck Cancer Patients. Cancers.

[12] Poni R., Neufeld E., Capstick M., Bodis S., Samaras T., Kuster N. (2021). Feasibility of Temperature Control by Electrical Impedance Tomography in Hyperthermia. Cancers.

[13] Androulakis I., Mestrom R.M.C., Christianen M.E.M.C., Kolkman-Deurloo I.-K.K., van Rhoon G.C. (2022). A Novel Framework for the Optimization of Simultaneous ThermoBrachyTherapy. Cancers.

[14] Scheidegger S., Mingo Barba S., Gaipl U.S. (2021). Theoretical Evaluation of the Impact of Hyperthermia in Combination with Radiation Therapy in an Artificial Immune—Tumor-Ecosystem. Cancers.

[15] Kok H.P., Crezee J. (2022). Fast Adaptive Temperature-Based Re-Optimization Strategies for On-Line Hot Spot Suppression during Locoregional Hyperthermia. Cancers.

